# Muscle and mind: rewiring cognitive-motor recovery through exercise-responsive neurophysiology in neurological populations

**DOI:** 10.3389/fpsyg.2026.1845070

**Published:** 2026-06-17

**Authors:** Andrea Calderone, Alessio Baricich, Sanaz Pournajaf, Fabrizio Sottile, Maria Grazia Maggio, Mirjam Bonanno, Rosaria De Luca, Francesco Ligato, Angelo Quartarone, Rocco Salvatore Calabrò

**Affiliations:** 1 IRCCS Centro Neurolesi Bonino Pulejo, Messina, Italy; 2 Department of Biomedical Sciences, Humanitas University, Milan, Italy; 3 IRCCS Humanitas Research Hospital, Milan, Italy; 4 Research Area in Neuromotor Rehabilitation and Rehabilitation Robotics, Department of Neuroscience, IRCSS San Raffaele Roma, Rome, Italy; 5 Department of Mental and Physical Health and Preventive Medicine, University of Campania ‘Luigi Vanvitelli’, Naples, Italy

**Keywords:** cognitive-motor integration, dual-task training, exercise, exerkines, neurological disorders, neuroplasticity, neurorehabilitation, virtual reality

## Abstract

Exercise in neurological rehabilitation is often prescribed to improve mobility, yet its influence extends to cognition through interacting peripheral, central, and behavioral pathways. This narrative review synthesizes evidence on how skeletal muscle activity can engage peripheral biomarkers and system-level interactions that become clinically meaningful only when translated into behaviorally relevant cognitive-motor integration (CMI). We examine neurotrophic, metabolic, immune, vascular, and endocrine relays, then consider central translation through synaptic plasticity, metabolic adaptation, neurovascular coupling, white matter integrity, and functional network reorganization. The central argument is that cognitive benefit cannot be inferred from biomarker change alone. Exercise is most likely to support recovery when biological pathway engagement is paired with attentionally demanding movement, dual-task control, gait automaticity, error-based adaptation, and context-sensitive learning. Research in stroke, Parkinson’s disease, multiple sclerosis, and traumatic brain injury illustrates the heterogeneous benefits of exercise. This heterogeneity may be shaped by lesion topology, inflammatory activity, fatigue, autonomic stability, medication state, baseline fitness, and exercise tolerance, all of which alter the internal biological dose generated by a given external training prescription. Virtual reality, robotics, wearable sensing, and non-invasive brain stimulation may strengthen this framework when they manipulate task complexity, salience, repetition, feedback, and ecological monitoring for a defined mechanistic purpose. Current evidence supports exercise as a biologically and behaviorally meaningful adjunct in neurorehabilitation, but it cautions against uniform prescriptions and overly direct biomarker interpretations. A more precise next generation of trials should integrate exercise prescription, mechanistic readouts, technology-enabled assessment, and ecologically valid cognitive-motor outcomes within the same translational logic. Across the review, CMI is treated as the behavioral test of whether exercise-responsive biology has been recruited into adaptive function, rather than as a separate concept appended after conditioning.

## 1 Introduction

Neurological rehabilitation has often been organized around a practical distinction between motor recovery and cognitive recovery. That separation is clinically convenient, but it is biologically and behaviorally incomplete. Everyday function depends on the continuous conversion of perception, attention, prediction, action selection, and error monitoring into adaptive movement. For example, walking in a crowded corridor while carrying an object, responding to a question, avoiding another person, and preparing to reach for a door handle requires simultaneous postural control, environmental monitoring, motor planning, and executive regulation rather than an isolated motor program.

In this review, cognitive-motor integration (CMI) is defined operationally as the capacity to coordinate cognitive control processes and motor execution so that goal-directed movement remains accurate, adaptable, and context-sensitive under changing environmental and internal demands. CMI is related to, but not identical with, dual-task interference, cognitive postural control, deliberate motor control, or simple movement automaticity. Dual-task interference describes the performance cost that arises when two tasks compete for resources; cognitive postural control refers more specifically to attentional regulation of balance; deliberate motor control denotes conscious supervision of movement; and automaticity refers to efficient execution with reduced attentional cost. CMI is broader because it concerns the flexible coupling between cognition and action across these contexts.

This distinction is clinically important because gait slowing, postural instability, reduced automaticity, impaired divided attention, and executive inefficiency frequently cluster in neurological disorders. In stroke, Parkinson’s disease (PD), multiple sclerosis (MS), and traumatic brain injury (TBI), cognitive dysfunction can compromise mobility, balance, motor learning, safety, and participation, while motor impairment can in turn increase cognitive load. The convergence between cognition and movement is therefore not incidental; it is a central feature of disability and recovery in neurorehabilitation (Xiang et al., 2022).

Exercise occupies a distinctive position within this framework. Contracting skeletal muscle is not merely the recipient of motor commands; it functions as a signaling organ that releases myokines and metabolic messengers, alters inflammatory and endocrine tone, engages vascular regulation, and may influence neurotrophic support (Pedersen and Febbraio, 2012; Kim et al., 2019; Blume and Royes, 2024; Kostka et al., 2024). The clinical relevance of those signals, however, depends on whether they are translated into central physiological states and then recruited during behaviorally meaningful practice. Exercise is therefore better understood as a biological exposure that may support CMI when paired with learning-relevant movement rather than as a simple conditioning tool for muscles and gait alone (Erickson et al., 2019; El-Sayes et al., 2019; Augusto-Oliveira et al., 2023).

The word neuroplasticity is often used to summarize this literature, but it becomes too imprecise when detached from mechanism. It does not specify which peripheral mediators are plausible, whether the evidence is human or preclinical, how vascular and metabolic constraints shape brain responsiveness, why some patients improve while others do not, or why biomarker shifts can occur without measurable cognitive gain. A mechanistic review must therefore distinguish pathway engagement from recovery, peripheral signaling from central translation, and neural change from ecologically useful behavior.

Disease context further reshapes the meaning of any exercise session. Stroke introduces lesion timing, vascular reserve, diaschisis, and task-specific relearning. PD brings dopamine-sensitive frontostriatal dysfunction, impaired automaticity, cueing dependence, and medication-state effects. MS adds inflammatory activity, demyelination, heat sensitivity, fatigue, and fluctuating disability. TBI introduces injury heterogeneity, autonomic instability, sleep disturbance, headache, vestibulo-ocular symptoms, and exertional tolerance limits. The same external workload can therefore generate different internal biological and cognitive-motor demands across patients.

**FIGURE 1:**
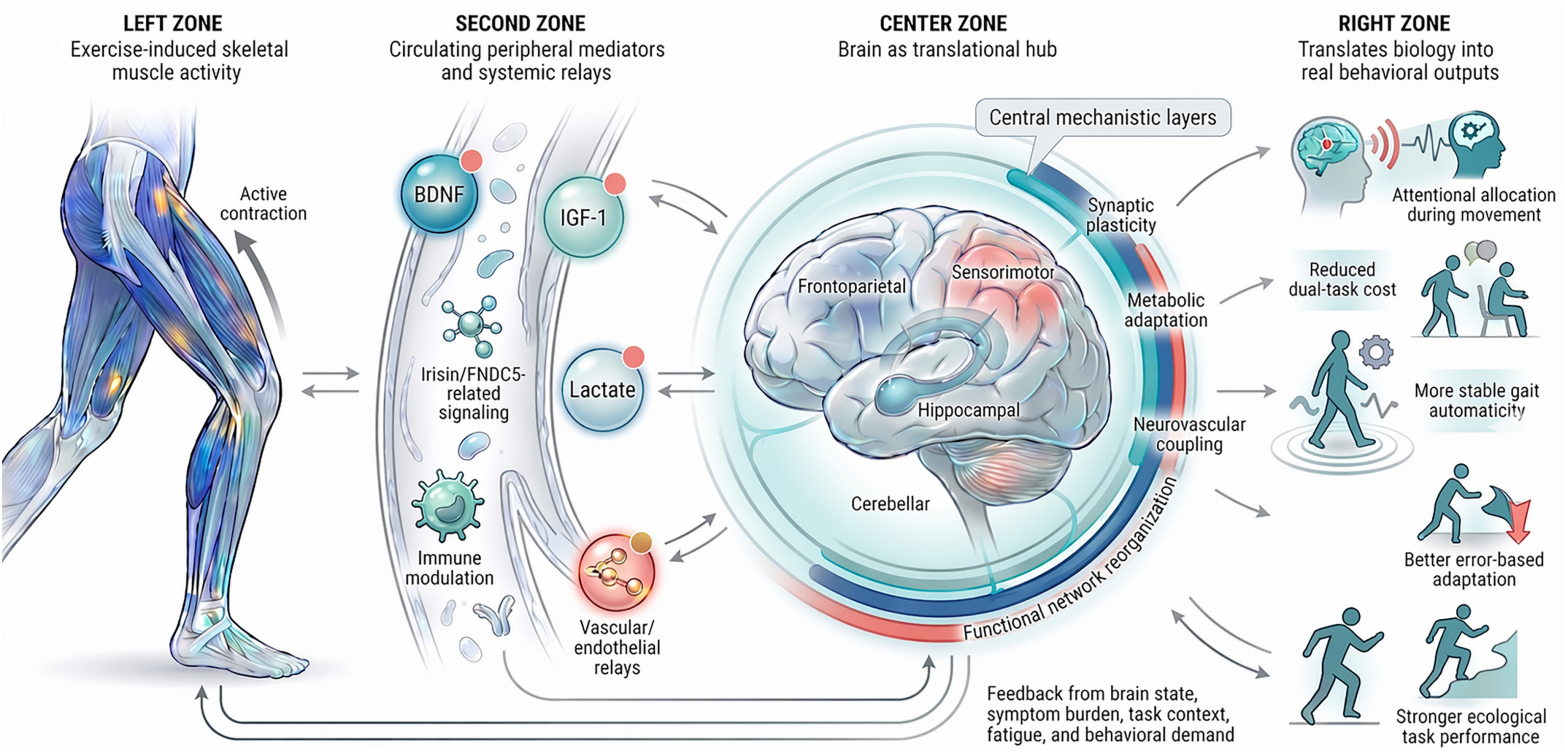
Integrated model of exercise-responsive cognitive-motor recovery. Schematic overview of the pathway linking skeletal muscle activity to cognitively meaningful neurorehabilitation outcomes. The figure begins with exercise-induced contraction and proceeds through circulating mediators, immune and vascular relays, and central translation mechanisms including synaptic plasticity, metabolic adaptation, neurovascular coupling, and network reorganization. Downstream outputs emphasize behavior rather than biomarker change alone, including improved attentional allocation, reduced dual-task cost, more stable gait automaticity, better error-based adaptation, and stronger performance during ecologically relevant tasks. Bidirectional arrows indicate that brain state, symptom burden, task context, fatigue, and behavioral demand also shape the biological meaning of a given exercise bout. The figure should be read as a translational sequence in which CMI is the behavioral endpoint of pathway engagement, not a separate downstream add-on.

The aim of this narrative review is to synthesize an integrative model linking exercise-responsive peripheral mediators, central neurophysiological translation, and the behavioral expression of CMI in neurorehabilitation. Particular attention is given to stroke, PD, MS, and TBI, because these conditions illustrate why cognitive benefit remains real yet heterogeneous. A second aim is to clarify how technology-enabled and personalized rehabilitation strategies can use exercise more precisely, not as a universal remedy, but as a targeted exposure whose value depends on internal load, task relevance, and disease-specific constraints. The overarching multiscale framework synthesized in this review is shown in Figure 1.

Accordingly, the organizing logic of this review is deliberately sequential: exercise first generates a peripheral and systemic biological exposure; this exposure may then be translated into central physiological readiness; and only when this readiness is challenged by attentionally demanding, goal-directed movement does it become visible as CMI.

This sequence also explains why CMI cannot be reduced to a generic dual-task label. Dual-task and gait-cognition literature shows that the cognitive operation loaded during movement can determine whether patients show motor-priority, cognitive-priority, or mutual-interference patterns, and those patterns differ across stroke, Parkinsonian gait, and broader neurological mobility disorders (Al-Yahya et al., 2011; Plummer et al., 2013; Yogev-Seligmann et al., 2008). Therefore, the mechanistic question is not only whether exercise changes biology, but whether the selected task exposes the disease-specific bottleneck through which biology can influence function.

## 2 Methods

This article was designed as a narrative review because the central question is integrative rather than purely aggregative. The goal was to connect biological, neurophysiological, behavioral, and clinical evidence that is distributed across different traditions, including exercise neuroscience, neurorehabilitation, movement science, and cognitive medicine. Narrative synthesis was therefore used to accommodate conceptual heterogeneity in populations, exercise modalities, biomarker timing, neuroimaging outcomes, and cognitive measures. The approach followed core recommendations for transparent biomedical narrative reviews, including explicit scope definition, clear search framing, stated evidence priorities, and direct acknowledgment of methodological limits (Gasparyan et al., 2011, Franco et al., 2018).

Literature identification was centered on PubMed/MEDLINE, PsycINFO, Embase, Scopus and was updated on 16 March 2026. Search clusters combined terms related to exercise and rehabilitation, including exercise, physical activity, aerobic training, resistance training, interval training, and dual-task training, with terms related to cognition and CMI, including cognition, executive function (EF), attention, working memory (WM), processing speed, cognitive-motor integration, and neurorehabilitation. Additional clusters targeted clinical populations, namely stroke, PD, MS, and TBI, and mechanistic domains such as brain-derived neurotrophic factor (BDNF), insulin-like growth factor 1 (IGF-1), irisin, fibronectin type III domain-containing protein 5 (FNDC5), lactate, exerkines, neuroinflammation, neurovascular coupling, cerebral blood flow, functional connectivity, white matter, virtual reality (VR), robotics, wearable sensors, and non-invasive brain stimulation (NIBS). Backward and forward citation tracking from key reviews and primary studies was used to refine topic coverage and update newer signals or technologies that were directly relevant to the narrative framework.

Reference selection also prioritized targeted mechanistic sources when they clarified a specific link in the translational chain, including lactate shuttle signaling, IGF-1-mediated exercise-brain effects, neurovascular-unit interpretation, disease-specific CMI, dual-task gait evidence, wearable ecological monitoring, and biomarker-surrogate endpoint logic.

Eligibility was determined by relevance to the review question rather than by a single study design hierarchy. Priority was given to recent systematic reviews, meta-analyses, randomized controlled trials, prospective intervention studies, mechanistic human experiments, and landmark translational studies that clarified biologically plausible links between exercise and cognitive or cognitive-motor outcomes. Preclinical work was incorporated when it materially strengthened mechanistic interpretation, especially for pathways such as FNDC5-irisin, hippocampal signaling, mitochondrial adaptation, and immune modulation that remain incompletely resolved in clinical populations. Preference was given to literature published from 2020 onward, although older seminal studies were retained when they defined a pathway that remains central to the field.

**FIGURE 2:**
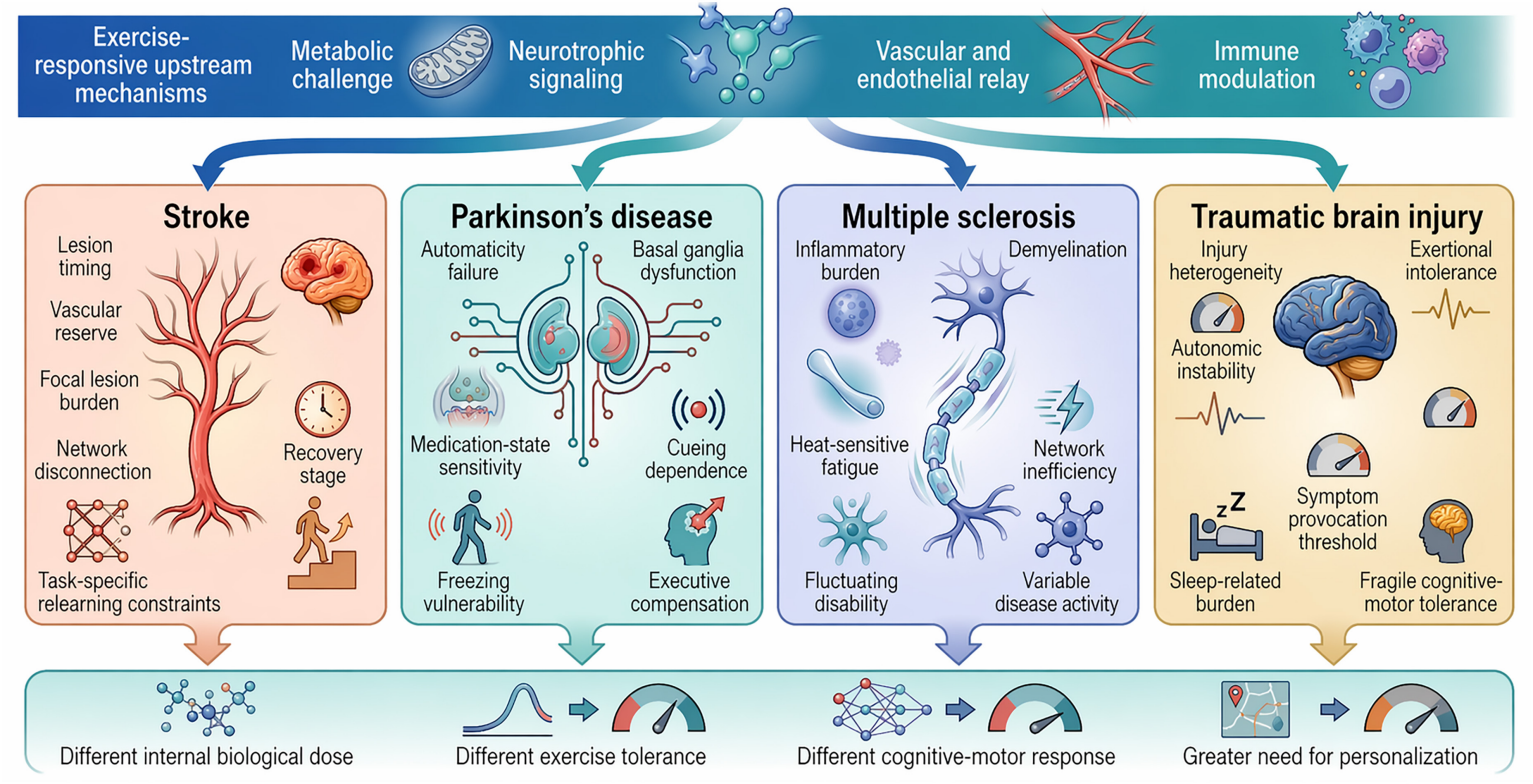
Disease-specific modifiers of exercise-responsive cognitive-motor integration. Conceptual comparison of how shared exercise-responsive pathways enter distinct pathological landscapes in stroke, Parkinson’s disease, multiple sclerosis, and traumatic brain injury. Shared upstream mechanisms include metabolic challenge, neurotrophic signaling, vascular and endothelial relay, and immune modulation. Disorder-specific nodes highlight lesion timing and vascular reserve in stroke, automaticity failure and cueing dependence in Parkinson’s disease, inflammatory burden and heat-sensitive fatigue in multiple sclerosis, and injury heterogeneity, autonomic instability, and symptom provocation threshold in traumatic brain injury. The common lower band emphasizes that nominally similar exercise prescriptions can yield different internal biological dose, exercise tolerance, cognitive-motor response, and need for personalization across conditions. The comparison emphasizes that similar external prescriptions can require different CMI task designs because each diagnosis constrains translation differently.

**FIGURE 3:**
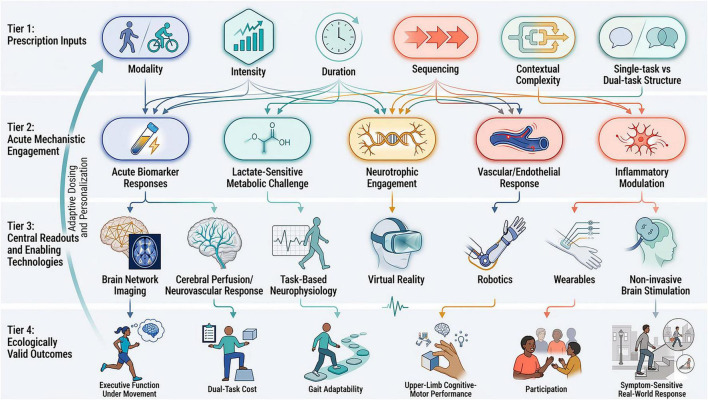
Translational roadmap for next-generation trials in cognitive-motor neurorehabilitation. Integrated framework linking exercise prescription to mechanistic engagement, central readouts, enabling technologies, and ecologically valid outcomes. Upstream components include modality, intensity, duration, sequencing, contextual complexity, and single-task versus dual-task structure. Intermediate layers include acute biomarker responses, lactate-sensitive metabolic challenge, neurotrophic engagement, vascular and endothelial response, and inflammatory modulation, followed by central readouts and enabling technologies such as brain network imaging, cerebral perfusion or neurovascular response, task-based neurophysiology, virtual reality, robotics, wearables, and non-invasive brain stimulation. Downstream outcomes include executive function under movement, dual-task cost, gait adaptability, upper-limb cognitive-motor performance, participation, and symptom-sensitive real-world function. The feedback loop indicates how early biological and behavioral signals can support adaptive dosing and personalization. The roadmap explicitly requires alignment among exercise dose, mechanistic marker, central readout, CMI task, and ecological outcome. Future trials should use this roadmap prospectively, so that each biomarker is assigned to a specific central readout and each central readout is assigned to a disease-specific CMI task.

**FIGURE 4:**
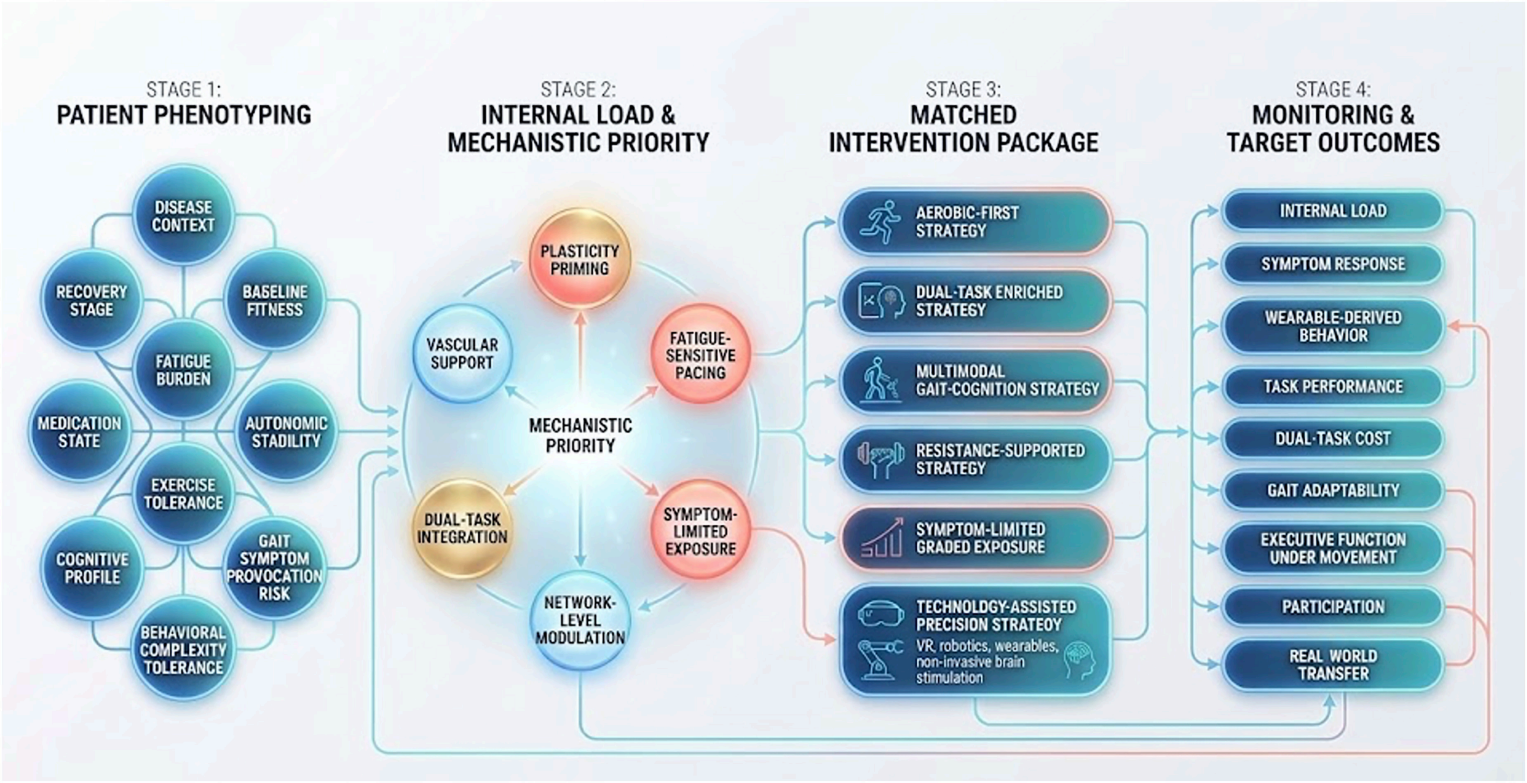
Precision phenotyping and adaptive prescription framework for cognitive-motor neurorehabilitation. Conceptual framework linking patient phenotyping to mechanistic priorities, matched intervention packages, and monitoring targets. The figure emphasizes how disease context, recovery stage, baseline fitness, fatigue burden, autonomic stability, exercise tolerance, symptom provocation risk, and cognitive profile can shape internal load and the selection of aerobic-first, dual-task enriched, multimodal gait-cognition, resistance-supported, symptom-limited, and technology-assisted strategies. Downstream monitoring integrates internal load, symptom response, wearable-derived behavior, task performance, dual-task cost, gait adaptability, executive function under movement, participation, and real-world transfer to support iterative personalization. The framework positions task complexity, symptom response, and real-world transfer as adaptive dosing signals for personalized CMI prescription.

For figure preparation, Gemini 3.1 Pro (Google DeepMind; source: Gemini App/Google AI Studio) was used under direct author supervision exclusively to support the creation and technical revision of Figures 1–4, including artifact removal and minor typographical or formatting corrections. No generative AI tool was used to generate scientific content, interpret the literature, select references, or formulate the manuscript’s arguments. All figure content and all manuscript content were critically reviewed, verified, and approved by the authors.

## 3 Peripheral biological mediators linking exercise to cognitive recovery

### 3.1 Myokines, neurotrophins, and metabolic messengers

A useful starting point for a muscle-brain model is the recognition that skeletal muscle behaves as a signaling organ during exercise rather than as a simple consumer of neural commands. Repeated contraction releases myokines and metabolic messengers, shifts endocrine tone, changes substrate availability, and modulates vascular and immune communication. These signals should be interpreted as coordinated relays rather than isolated molecules, because tissue source, sampling compartment, acute timing, training status, and disease biology strongly shape their meaning (Pedersen and Febbraio, 2012; Kim et al., 2019; Blume and Royes, 2024; Kostka et al., 2024).

Within the present CMI framework, these mediators are not interpreted as isolated causes of cognitive recovery. They are better viewed as components of an internal biological dose that may raise or lower the probability that a patient can engage in cognitively demanding movement, tolerate error, update an action plan, and consolidate task-relevant practice. Their clinical significance therefore depends on whether they are temporally and behaviorally coupled to CMI rather than measured as detached laboratory signals.

BDNF remains the most discussed mediator because it supports synaptic maintenance, dendritic remodeling, long-term potentiation, and activity-dependent learning. Acute exercise can increase circulating BDNF, and exercise training has been associated with cognitive and structural brain outcomes, including hippocampal changes in older adults (Gomez-Pinilla et al., 2008; Erickson et al., 2011; Mackay et al., 2017; Erickson et al., 2019). In stroke survivors, exercise-related BDNF responses are detectable but vary with exercise modality, sampling schedule, chronicity, and baseline disability (Ashcroft et al., 2022; Khalil, 2025). In PD, exercise-related BDNF changes have been examined in relation to clinical and motor-cognitive outcomes, but effects remain moderated by medication state, disease stage, modality, and dose (Zoladz et al., 2014; Kaagman et al., 2024). Evidence in MS and TBI is more heterogeneous and should be interpreted as pathway-relevant rather than as proof of repair.

Peripheral BDNF is therefore informative but not definitive (Edman et al., 2024). Serum or plasma concentrations can reflect release from platelets, vascular endothelium, immune cells, skeletal muscle, and brain-related sources, and they cannot be treated as a direct readout of central repair. A stronger interpretation is that BDNF may index engagement of a plasticity-relevant systemic pathway whose clinical meaning depends on whether central physiology and behavioral practice are aligned with that signal.

The FNDC5-irisin axis illustrates both the appeal and the uncertainty of exercise-responsive myokines. Experimental work suggests that exercise-induced peroxisome proliferator-activated receptor gamma coactivator 1-alpha (PGC-1α)/FNDC5 signaling can support hippocampal BDNF expression and memory-related adaptation (Wrann et al., 2013; Islam et al., 2021). However, human interpretation remains limited by assay specificity, tissue origin, acute kinetics, and disease-specific biology (Pignataro et al., 2021; Sadier et al., 2024; Paoletti and Coccurello, 2024). Irisin should therefore be presented as a biologically plausible mediator rather than as a validated clinical biomarker of cognitive recovery in stroke, PD, MS, or TBI.

IGF-1 provides a complementary endocrine route. Exercise can alter circulating IGF-1, and IGF-1 can cross the blood-brain barrier and interact with BDNF-related signaling, synaptic regulation, cell survival, and metabolic adaptation (Ding et al., 2006; Torres-Aleman, 2010; Yan et al., 2011; Zegarra-Valdivia et al., 2025). This interaction is important because it prevents a single-molecule interpretation of exercise-induced plasticity. Neurotrophic effects are more plausibly expressed through converging pathways whose magnitude depends on age, endocrine state, training history, and disease context.

For neurological populations, the translational relevance of IGF-1 is likely strongest when it is considered as a permissive endocrine-metabolic signal that can interact with learning demand rather than as a stand-alone marker of neural repair. In stroke, IGF-1-sensitive synaptic and metabolic support may be most relevant when patients are exposed to task-specific relearning after vascular injury and diaschisis. In PD, its relevance is likely conditioned by dopaminergic state, frontostriatal reserve, and the capacity to recruit compensatory cognitive control during gait. In MS, endocrine-metabolic support may influence fatigue tolerance and the ability to sustain processing-speed-dependent practice, whereas in TBI it may interact with sleep, autonomic stability, and graded exertional tolerance. These disease-specific interpretations remain mechanistically plausible rather than conclusively proven, but they clarify why the same circulating signal may have different CMI implications across diagnoses (Carro et al., 2000; Carro et al., 2001; Cotman et al., 2007).

The most useful clinical interpretation is therefore not that higher IGF-1 equals recovery, but that IGF-1-related signaling may contribute to a biological window in which practice is more likely to be learned. A CMI-oriented trial would need to pair IGF-1 measurement with a central readout and a behavioral endpoint such as dual-task gait, adaptive reaching, visuomotor updating, or sustained executive control during movement before claiming that this endocrine pathway has become functionally meaningful.

Experimental evidence that circulating IGF-1 can mediate exercise-induced increases in hippocampal neurogenesis supports IGF-1 as a plausible exercise-brain relay, but this evidence remains closer to pathway plausibility than clinical proof in stroke, PD, MS, or TBI (Trejo et al., 2001). In a rehabilitation trial, the stronger design would test whether IGF-1 change covaries with a central readout and with retained CMI learning, rather than with immediate post-session performance alone.

Lactate has also been reinterpreted in exercise neuroscience. It is not merely a by-product of anaerobic metabolism; it is a transportable fuel and signaling molecule that crosses the blood-brain barrier via monocarboxylate transporters and can interact with redox state, sirtuin 1 (SIRT1)-dependent pathways, and BDNF-associated signaling (El Hayek et al., 2019; Müller et al., 2020; Lei et al., 2024). Its translational value lies in marking a metabolically meaningful internal challenge, but higher lactate is not inherently better. In neurological populations, fatigue, deconditioning, autonomic disturbance, gait inefficiency, or heat sensitivity can generate substantial internal stress at relatively low external workloads.

Lactate is particularly relevant to CMI because cognitively loaded movement imposes simultaneous energetic demands on contracting muscle, autonomic regulation, and distributed neural systems that support attention, inhibition, prediction, and error correction. A walking task that includes obstacle negotiation, verbal fluency, set shifting, or response inhibition may therefore change the internal metabolic meaning of an otherwise similar external workload. In this sense, lactate can help index whether the exercise-CMI stimulus has reached a metabolically meaningful challenge, but it cannot identify by itself whether the challenge was adaptive, excessive, or behaviorally useful.

The lactate-shuttle framework further supports lactate as both substrate and signal, and HCAR1-related experimental work links exercise-associated lactate signaling to cerebral VEGF and angiogenic pathways (Brooks, 2018; Morland et al., 2017). For CMI, this matters because the same walking speed can generate a different internal metabolic signal when the patient must scan, inhibit, remember, or switch rules; lactate should therefore be interpreted as a contextual load marker, not as a universal therapeutic target (Quistorff et al., 2008; Bergersen, 2015; Skriver et al., 2014).

This distinction is clinically important in neurological rehabilitation. In stroke, elevated internal metabolic load may reflect deconditioning, inefficient gait, or the energetic cost of relearning. In PD, lactate dynamics may be shaped by bradykinesia, rigidity, medication state, and compensatory attentional control. In MS, a lactate-generating workload may quickly intersect with fatigue and heat sensitivity, whereas in TBI it may interact with autonomic instability, headache, dizziness, sleep disruption, and symptom provocation. Thus, lactate-responsive signaling is best interpreted together with perceived exertion, symptom response, movement quality, and post-exercise recovery when the therapeutic target is CMI.

The term threshold is therefore used here in a physiological, not absolute, sense. It refers to the point at which a given exercise exposure generates sufficient internal load to engage a target pathway, such as lactate-responsive signaling, BDNF release, vascular shear stress, or immune-endocrine modulation. This threshold is person- and disease-specific rather than fixed. It should be assessed through convergent indicators, including heart rate, perceived exertion, lactate when relevant, symptom response, movement quality, and post-exercise recovery, rather than through a single biomarker cutoff (Garber et al., 2011; Griesbach, 2011; Silverberg et al., 2020).

A practical implication is that biomarker thresholds should be aligned with behavioral thresholds. A session that engages lactate-responsive or neurotrophic signaling but causes attentional collapse, unsafe gait variability, or next-day symptom worsening is unlikely to be an effective CMI stimulus. Conversely, a moderate physiological challenge that preserves task accuracy, feedback use, and adaptive movement may be more clinically valuable than a larger biomarker response that cannot be translated into learning.

### 3.2 Immune, vascular, and endocrine relays from muscle to brain

Peripheral biology extends beyond classic growth factors and myokines (Table 1). Exercise also remodels immune, vascular, and endocrine environments that influence the conditions under which cognition is expressed. In neurological populations, these relays are especially relevant because inflammatory activity, endothelial dysfunction, altered cerebrovascular reserve, metabolic inflexibility, sleep disturbance, autonomic dysregulation, and fatigue can constrain the translation from practice to learning (Ribarič, 2022; Su et al., 2025; Su and Su, 2025).

**TABLE 1:** Exercise-responsive peripheral mediators linking skeletal muscle activity to cognition in neurological populations.

Peripheral mediator or relay	Primary exercise trigger	Main route or systemic relay to the brain	Principal neurophysiological relevance	Most plausible cognitive relevance	Representative neurological contexts	Major interpretive caveats
BDNF	Aerobic work, interval work, repeated training, metabolically challenging sessions	Systemic circulation, platelet release, endothelial interaction, partial BBB relevance	Supports synaptic plasticity, dendritic remodeling, and long-term potentiation (Gomez-Pinilla et al., 2008; Ashcroft et al., 2022).	Learning readiness, EF, memory, adaptive cognitive-motor performance	Stroke, PD, MS, TBI	Peripheral levels are tissue-non-specific, acute kinetics vary, and the marker is not a direct surrogate of brain repair (Mackay et al., 2017; Khalil, 2025).
FNDC5-irisin signaling	Sustained muscle contraction, PGC-1α related transcriptional activation	Circulating myokine signaling, possible BBB interaction, indirect hippocampal effects	May support BDNF-related signaling, neuroprotection, and metabolic resilience (Wrann et al., 2013; Islam et al., 2021).	Memory support, cognitive resilience, behaviorally enriched recovery	Stroke, PD, MS, TBI, broader neurodegeneration models	Human assay specificity and tissue origin remain uncertain, and clinical causality is incomplete (Pignataro et al., 2021; Sadier et al., 2024).
IGF-1	Aerobic and resistance exercise, training-induced anabolic adaptation	Circulation with BBB transport and peripheral-central endocrine crosstalk	Supports synaptic signaling, cell survival, and metabolic adaptation (Ding et al., 2006; Torres-Aleman, 2010). CMI relevance: may support learning readiness when paired with adaptive practice under cognitive-motor demand.	Learning, attentionally demanding performance, recovery support	Stroke, PD, age-related cognitive decline, mixed neurological populations Disease-specific interpretation is required; IGF-1 is not a direct CMI or recovery surrogate without central and behavioral linkage.	Responses depend on age, endocrine status, timing, and exercise dose (Yan et al., 2011; Zegarra-Valdivia et al., 2025).
Lactate	Higher internal metabolic load, interval work, vigorous bouts, deconditioned effort at lower workloads	Transport across BBB via monocarboxylate transporters, systemic metabolic signaling	Acts as an alternative brain fuel and redox-sensitive signal that may support plasticity-related pathways (El Hayek et al., 2019; Müller et al., 2020). CMI relevance: helps interpret internal metabolic challenge during dual-task or cognitively loaded movement.	Acute attentional and memory-related priming, threshold marker for cognitively relevant challenge	Stroke, PD, TBI, fatigue-limited conditions In MS and TBI, fatigue, heat sensitivity, autonomic symptoms, and symptom provocation may make high lactate maladaptive rather than beneficial.	Higher values are not inherently better, and internal dose differs across diseases and symptom states (Müller et al., 2020; Lei et al., 2024).
Immune mediators and inflammatory shift	Repeated training, moderate to vigorous exercise, habitual activity	Systemic cytokine profile, immune-endothelial interaction, microglial relevance	May reduce neurotoxic inflammatory tone and support repair-permissive conditions (Lozinski and Yong, 2022; Su et al., 2025).	Reduced cognitive fatigue, better consistency of cognitive-motor performance	MS, TBI, stroke, broader neurodegenerative conditions	Responses are diagnosis-specific, medication-sensitive, and highly time-dependent (Sanaeifar et al., 2024; Su et al., 2025).
Vascular and endothelial relays	Shear stress, increased cardiac output, repeated aerobic exposure	Endothelial nitric oxide signaling, arterial compliance, cerebrovascular responsiveness	Improves perfusion support and neurovascular coupling conditions (Claassen et al., 2021; Bliss et al., 2021).	Attention, processing efficiency, complex walking and task stability	Stroke, PD, cerebrovascular burden, mixed neurological populations	Perfusion changes do not guarantee behavioral gain and can be limited by vascular pathology (Tomoto et al., 2021; Tomoto et al., 2023).
Metabolic and endocrine milieu	Repeated training, improved fitness, resistance and aerobic adaptation	Systemic changes in insulin sensitivity, substrate use, mitochondrial efficiency	Supports energetic flexibility of neural tissue and whole-body tolerance to training (Umegaki et al., 2021; Tari et al., 2025).	Reduced cognitive fatigability, improved tolerance of sustained dual-task work	MS, TBI, chronic stroke, deconditioned neurological populations CMI relevance: may improve the capacity to sustain attentionally demanding movement without excessive fatigability.	This is a highly indirect pathway and is difficult to isolate from mood, sleep, and fitness effects (Ribarič, 2022; Tari et al., 2025).

BBB, blood-brain barrier; BDNF, brain-derived neurotrophic factor; EF, executive function; FNDC5, fibronectin type III domain-containing protein 5; IGF-1, insulin-like growth factor 1; MS, multiple sclerosis; PD, Parkinson’s disease; PGC-1α, peroxisome proliferator-activated receptor gamma coactivator 1-alpha; TBI, traumatic brain injury.

The immune effects of exercise should be described with compartmental precision. In humans, the most defensible claim is that regular exercise can modulate peripheral immune tone by altering circulating cytokine balance, reducing chronic low-grade inflammatory signaling in some contexts, increasing anti-inflammatory mediators after repeated training, and improving immune-metabolic regulation (Petersen and Pedersen, 2005; Pedersen and Febbraio, 2012). Interleukin-6 (IL-6) is especially important because contracting muscle releases IL-6 as a myokine during exercise, and this acute signal can stimulate anti-inflammatory mediators such as interleukin-10 (IL-10) and IL-1 receptor antagonist while inhibiting tumor necrosis factor-α under some conditions. By contrast, central neuroinflammatory change in humans remains harder to demonstrate directly and is usually inferred from preclinical models, neuroimaging proxies, cerebrospinal fluid markers, or disease-specific indirect evidence. This distinction is particularly important in MS and TBI, where inflammatory activity and recovery trajectories can fluctuate, and in PD, where peripheral inflammation and neuroimmune activation may interact with neurodegenerative progression and fatigue.

Vascular biology provides a second major relay. Contractile activity increases cardiac output and shear stress, supports endothelial nitric oxide signaling, and can increase cerebral blood flow during exercise while influencing arterial stiffness, cerebrovascular reactivity, and regional perfusion (Smith and Ainslie, 2017; Claassen et al., 2021; Bliss et al., 2021; Tomoto et al., 2021; Tomoto et al., 2023). These processes matter because cognition depends on timely coupling between neural demand and vascular supply. Nitric oxide signaling is also mechanistically relevant to neurotrophic regulation, because experimental and translational evidence indicates that nitric oxide synthesis and cerebrovascular endothelium-derived nitric oxide can participate in exercise-related BDNF regulation (Chen et al., 2006; Banoujaafar et al., 2016; Cefis et al., 2023). In stroke, vascular reserve and lesion territory can constrain the benefit of exercise; in PD, autonomic and vascular regulation may interact with gait automaticity and cognition; in MS, inflammatory and endothelial changes may influence fatigue and processing speed; and after TBI, cerebrovascular regulation and symptom provocation may shape safe progression.

Endocrine and metabolic context further shapes this relay. Exercise-sensitive changes in insulin action, substrate utilization, ketone availability, and mitochondrial resilience may influence the energetic cost of cognitively demanding movement, especially when disease-related network damage, perfusion constraints, or fatigue increase the cost of task performance (Sleiman et al., 2016; Umegaki et al., 2021; Tari et al., 2025). This pathway should be interpreted cautiously. Direct evidence that exercise training produces cerebral mitochondrial adaptation in human neurorehabilitation remains limited, and many clinical inferences rely on indirect markers such as fitness, fatigue, perfusion, and task performance. Peripheral signals are therefore best interpreted as a coordinated biological state whose cognitive relevance depends on central translation, timing, disease context, and behavioral embedding rather than on any one laboratory value.

The endocrine-metabolic relay is therefore disease-specific. After stroke, reduced vascular reserve, diaschisis, sarcopenia, and deconditioning can increase the metabolic cost of relearning even during apparently simple gait or reaching tasks. In PD, autonomic dysfunction, fatigue, dopamine-sensitive motor-cognitive circuitry, and medication-state fluctuations may determine whether exercise improves automaticity or merely increases compensatory cognitive load. In MS, inflammatory activity, demyelination, heat sensitivity, and impaired processing speed can make the same exercise dose either enabling or fatiguing. In TBI, sleep disturbance, headache, vestibulo-ocular symptoms, autonomic instability, and exertional intolerance can transform a conventional aerobic prescription into a symptom-provoking exposure unless progression is carefully graded.

These relays do not produce CMI directly. Rather, they shape whether CMI training is biologically tolerable, cognitively sustainable, and capable of transfer. Improved insulin action, substrate flexibility, vascular delivery, and recovery biology may allow a patient to maintain attention during walking, preserve movement quality while distracted, and repeat complex practice without excessive fatigue. Their value is therefore best evaluated by combining physiological monitoring with task-specific CMI outcomes instead of assuming that endocrine or metabolic improvement automatically constitutes cognitive recovery.

A key translational implication is that endocrine-metabolic markers should be modeled as modifiers of capacity rather than as linear predictors of cognition. A patient may show improved fitness or substrate regulation yet still fail a complex walking task if neurovascular reserve, fatigue, autonomic tolerance, or attentional control remains limiting. This is why endocrine-metabolic outcomes should be paired with CMI endpoints that preserve both motor quality and cognitive accuracy (Gleeson et al., 2011; Motl et al., 2017; Saunders et al., 2020).

## 4 Central neurophysiological translation of exercise-related signals

### 4.1 Synaptic plasticity, neurogenesis-related signaling, and metabolic adaptation

Peripheral signals matter in neurorehabilitation only if they are translated into central changes that alter cognition or CMI. The strongest candidate mechanisms at this level involve synaptic plasticity, cellular energetics, neurogenesis-related signaling, neurovascular support, and network efficiency (Table 2). Exercise should not be described as repairing the brain uniformly; rather, it may increase the probability that adaptive neural change becomes possible when appropriate behavioral demands are imposed (El-Sayes et al., 2019; de Sousa Fernandes et al., 2020; Augusto-Oliveira et al., 2023).

**TABLE 2:** Central neurophysiological mechanisms through which exercise may influence cognition and cognitive-motor function.

Central mechanism	Key upstream exercise-related drivers	Representative human support	Representative preclinical support	Most plausible cognitive or cognitive-motor consequences	Main uncertainties or limitations
Synaptic plasticity and long-term potentiation	BDNF, IGF-1, activity-dependent signaling, repeated practice	Biomarker studies, rehabilitation trials, and indirect human neurophysiology support this mechanism (El-Sayes et al., 2019; Erickson et al., 2019).	Strong mechanistic support has been shown in exercise and learning models (Gomez-Pinilla et al., 2008; Ding et al., 2006).	Improved EF, learning efficiency, adaptive task performance	Human causal pathways remain indirect and biomarker interpretation is imperfect
Hippocampal plasticity and memory-related signaling	FNDC5-irisin related pathways, BDNF, metabolic support	Associations with hippocampal volume, perfusion, and memory-sensitive outcomes have been reported in humans (Aghjayan et al., 2021; Karamacoska et al., 2023).	Robust experimental support exists for exercise-sensitive hippocampal adaptation (Wrann et al., 2013; Islam et al., 2021).	Memory support, contextual learning, cognitive resilience	Direct human evidence for neurogenesis remains limited
Mitochondrial and metabolic adaptation	Lactate signaling, improved substrate handling, repeated aerobic challenge	Indirect human evidence comes from fitness, fatigue, and exercise-cognition studies (Müller et al., 2020; Tari et al., 2025).	Experimental work supports improved energetic flexibility and cellular resilience (Dietrich et al., 2008; Lei et al., 2024).	Reduced cognitive fatigability, more efficient neural recruitment	Translational markers are still non-specific and often inferential
Neurovascular coupling and cerebrovascular responsiveness	Shear stress, endothelial adaptation, improved cardiovascular function	Cerebral blood flow and vascular-function studies support this pathway in aging and neurological contexts (Claassen et al., 2021; Bliss et al., 2021; Tomoto et al., 2023).	There is a consistent physiological rationale for coupling neural demand to perfusion (Claassen et al., 2021; Tomoto et al., 2021).	Better attention, processing efficiency, stability under cognitive-motor load	Perfusion effects vary by disease stage, vascular burden, and intervention dose Interpretation should be disease-specific: focal vascular injury in stroke, frontostriatal-cueing demands in PD, inflammatory/demyelinating constraints in MS, and cerebrovascular/autonomic dysregulation in TBI.
White matter and myelin-sensitive remodeling	Reduced inflammatory burden, repeated movement, metabolic support	Diffusion-sensitive rehabilitation studies, especially in MS and stroke, are compatible with this mechanism (Prosperini et al., 2014).	Translational support is plausible, although the specific repair substrate remains uncertain (Prosperini and Di Filippo, 2019; de Sousa Fernandes et al., 2020).	Improved signal transmission, reduced dual-task interference, better network efficiency CMI relevance includes long-range communication for dual-task gait, adaptive reaching, processing speed, and attentional control during movement.	Imaging markers are indirect and not specific to one repair process Relevant substrates differ across MS demyelination, TBI diffuse axonal injury, stroke tract integrity, and PD basal ganglia-cortical communication; imaging markers require behavioral confirmation.
Large-scale network reorganization	Fitness gains, repeated cognitive-motor challenge, multisystem engagement	Functional connectivity and task-based imaging reviews support this pathway in humans (van der Stouwe et al., 2018; Won et al., 2021; Smith et al., 2025).	Systems-level experimental and translational work supports network adaptation with training (de Sousa Fernandes et al., 2020; Augusto-Oliveira et al., 2023).	Improved attentional allocation, planning, gait-cognition coordination, adaptive control	Network findings are heterogeneous and often not tightly linked to ecological outcomes Network change should be interpreted through ecologically valid CMI outcomes rather than global cognitive screening alone.

BDNF, brain-derived neurotrophic factor; EF, executive function; IGF-1, insulin-like growth factor 1; MS, multiple sclerosis.

This translation step is where disease heterogeneity becomes decisive. The same peripheral signal may encounter an ischemic network with impaired perfusion reserve after stroke, dopamine-sensitive frontostriatal dysfunction in PD, demyelinated and fatigue-sensitive pathways in MS, or diffuse axonal and autonomic disruption after TBI. Consequently, central responsiveness cannot be inferred from peripheral pathway engagement alone; it must be tested through neurophysiology, perfusion or network measures, and CMI behavior that reflects the affected disease system.

Synaptic plasticity remains the conceptual core of most exercise-cognition models, but the human evidence is strongest when biomarker findings are paired with neurophysiological measures. TMS studies using paired associative stimulation indicate that a single bout of aerobic exercise can facilitate long term potentiation (LTP)-like motor cortical plasticity in humans (McDonnell et al., 2013; Singh et al., 2014; Mang et al., 2014). These findings support the claim that exercise can transiently prime plasticity-related neurophysiology, but they do not by themselves demonstrate cognitive recovery. Their value is that they provide a measurable bridge between peripheral exercise exposure, central excitability, and learning-relevant practice (Skriver et al., 2014; Statton et al., 2015; Cramer et al., 2011).

For CMI, the key issue is whether exercise-induced plasticity readiness is captured during the correct behavioral window. A bout of aerobic exercise may transiently alter excitability or LTP-like responsiveness, but the functional target differs across diseases: visuospatial and error-based relearning after stroke, cue-dependent gait control in PD, fatigue-sensitive balance-attention coupling in MS, and graded attention under exertion after TBI. The same neurophysiological priming signal may therefore require different task pairings to become clinically meaningful.

Adult neurogenesis-related signaling should be distinguished from synaptic plasticity and hippocampal physiology. Exercise has been associated with hippocampal volume, perfusion, memory-related function, and learning-sensitive network changes in humans, while direct causal inference about adult human neurogenesis remains limited (Erickson et al., 2011; Aghjayan et al., 2021; Karamacoska et al., 2023). The conservative interpretation is that exercise may influence hippocampal physiology, metabolic support, and memory-relevant networks even when the exact cellular substrate cannot be measured directly in clinical populations (van Praag et al., 1999; Pereira et al., 2007; Maass et al., 2015).

Central translation also depends on energetics. In neurological disease, cognitively demanding behavior may fail because damaged circuitry, reduced vascular reserve, fatigue, autonomic dysregulation, or impaired substrate handling increases the cost of neural recruitment. Rather than invoking a vague “favorable metabolic milieu,” the relevant mechanisms include lactate transport, insulin and substrate regulation, ketone-related BDNF signaling, vascular delivery, and mitochondrial resilience. Evidence for these pathways is strong in preclinical and translational work, whereas direct evidence of cerebral mitochondrial adaptation to exercise training in human neurorehabilitation remains limited (Dietrich et al., 2008; Müller et al., 2020; Sleiman et al., 2016; Tari et al., 2025).

Energetic translation is also a CMI problem because complex movement requires sustained neural recruitment while the body is simultaneously managing exercise load. A patient who can cycle automatically may still fail when walking while inhibiting a response, turning while processing a cue, or reaching while updating a plan. This discrepancy supports the need to measure metabolic adaptation together with cognitive-motor performance rather than treating improved fitness as a sufficient proxy for improved cognitive control during movement.

These mechanisms should not be idealized. Biomarker shifts may occur without measurable cognitive gain because the sampling window may not match the behavioral window, the outcome may be too global to capture the targeted process, the exercise dose may engage a pathway without sufficient task-specific practice, or disease constraints may prevent translation into behavior. Conversely, functional improvement can occur without a clear peripheral biomarker signature if the primary effect is task learning, compensatory strategy use, or improved ecological tolerance. The mismatch is therefore informative: it indicates that central translation depends on lesion burden, disease timing, medication state, baseline fitness, outcome selection, and behavioral context (Fleming and Powers, 2012; Cramer et al., 2011; Dobkin, 2009).

### 4.2 Neurovascular coupling, network reorganization, and systems-level cognition

A systems-level view becomes essential once the question moves from molecular plausibility to real-world cognition. Neurovascular coupling refers to the capacity of local vascular responses to match regional neural metabolic demand. It is related to, but not identical with, functional connectivity. Blood oxygen level-dependent (BOLD)-based functional connectivity partly depends on vascular physiology, whereas functional connectivity describes correlated activity across distributed regions. This distinction matters because an exercise intervention may improve vascular responsiveness without necessarily reorganizing cognitive networks, or may alter task-related activation without proving vascular repair (Smith and Ainslie, 2017; Claassen et al., 2021; Bliss et al., 2021).

Neurovascular coupling also has different meanings across neurological populations. In stroke, the relevant question is how focal vascular injury, lesion territory, collateral reserve, and peri-lesional perfusion influence the ability to relearn movement under cognitive load. In MS, vascular and inflammatory changes interact with demyelination, fatigue, and processing-speed limitations rather than with a single focal infarct. In PD, neurovascular support must be considered alongside dopamine-sensitive basal ganglia-cortical and frontostriatal networks that regulate automaticity, cue responsiveness, and executive control during gait. In TBI, diffuse network disruption, altered cerebrovascular regulation, sleep disturbance, autonomic instability, and symptom provocation can limit the safe translation of exercise into attention and processing-speed gains.

This disease-specific framing prevents neurovascular change from being treated as a generic marker of brain health. A perfusion or cerebrovascular response becomes clinically meaningful only if it supports the task demands that are impaired in the patient, such as divided attention during walking after stroke, cue-triggered gait adaptation in PD, fatigue-resistant balance control in MS, or symptom-limited cognitive exertion after TBI (Ogoh and Ainslie, 2009; Willie et al., 2014; Maass et al., 2015).

The neurovascular-unit framework reinforces this caution because functional hyperemia depends on coordinated vascular, glial, neuronal, and pericytic responses rather than on blood flow alone (Iadecola, 2017). In a CMI trial, neurovascular readouts are most informative when they are linked to the task that stresses the relevant network, such as turning under inhibition in PD, obstacle avoidance after stroke, balance-attention coupling in MS, or exertional attention after TBI.

Large-scale network reorganization provides a second pathway. Neuroimaging reviews suggest that exercise and cardiorespiratory fitness are associated with changes in functional connectivity, gray matter preservation, and network efficiency (Won et al., 2021; Smith et al., 2025). In PD, this may involve frontostriatal and sensorimotor-cognitive circuits that support automaticity and cue responsiveness. After TBI, altered cerebrovascular regulation, diffuse network disruption, sleep disturbance, and symptom provocation can influence how exercise affects attention and processing speed. In stroke and MS, lesion topology, vascular reserve, demyelination, and inflammatory activity can all determine whether network-level change becomes behaviorally useful.

White matter and myelin-sensitive mechanisms remain especially relevant in MS and diffuse injury states, but they should be interpreted cautiously. Rehabilitation and exercise have been linked to diffusion- and connectivity-sensitive changes compatible with improved network communication, particularly in MS, yet diffusion markers are not specific to remyelination, axonal repair, edema, or inflammation (Prosperini et al., 2014; Prosperini and Di Filippo, 2019). These findings support the broader principle that exercise may influence communication infrastructure, but they do not identify a single repair substrate.

White matter interpretation should likewise be broadened beyond MS while remaining cautious. In TBI, diffuse axonal injury can disrupt long-range communication required for attention, processing speed, and dual-task coordination. In stroke, corticospinal and association-tract integrity may influence diaschisis, compensatory recruitment, and the ability to integrate cognition with paretic movement. In PD, basal ganglia-cortical and frontoparietal communication may condition whether patients can shift from automatic gait to deliberate cue-based control. In MS, demyelination and possible remyelination are central, but diffusion-sensitive changes still cannot be equated with a specific repair process without convergent biological and behavioral evidence.

Diffusion and connectivity measures should therefore be treated as communication-readiness markers. They can identify whether long-range pathways are structurally or functionally available for CMI, but they do not specify whether the patient can use those pathways during a real task. Behavioral confirmation remains essential because improved tract integrity, connectivity, or network efficiency may still fail to transfer if the selected task overloads attention, fatigue tolerance, or safety (Sexton et al., 2016; Taubert et al., 2010; Cramer et al., 2011).

The network view also clarifies why combined cognitive-motor interventions may outperform unimodal exercise for selected outcomes. If the relevant target is not generic fitness but coordination of attention, prediction, action updating, and sensory integration, then exercise alone may be insufficient. The biological state produced by exercise may need to coincide with cognitively loaded movement, explicit adaptation, or ecologically valid practice before measurable CMI benefit appears. Dual-task and multicomponent reviews support this interpretation, although specific protocols remain heterogeneous (Fritz et al., 2015; Leone et al., 2017; Chartier et al., 2024).

This systems-level perspective helps reconcile a persistent tension in the field. Exercise can be biologically active while producing modest clinical cognitive effects if the outcome does not capture the process most likely to change. Improved attentional stability during walking, reduced cognitive-motor interference, faster online correction, or greater tolerance of contextual complexity may not be fully reflected in brief global cognitive screening. Network-level change, vascular support, and behavioral ecology therefore need to be measured together.

For this reason, CMI tasks can serve as a behavioral stress test of central translation. If exercise improves a vascular, metabolic, or network marker but the patient remains unable to divide attention, adapt gait, use feedback, or maintain movement quality under distraction, the biological change has not yet been shown to support the target function. Conversely, improved CMI performance can identify clinically useful translation even when a single biomarker does not change in a predictable direction.

## 5 Cognitive-motor integration as the behavioral expression of exercise-induced plasticity

### 5.1 Automaticity, attentional allocation, and dual-task control

CMI is the behavioral level at which biological readiness becomes functionally meaningful. It is expressed when a patient must walk while responding to information, negotiate obstacles, time a reach, divide attention, correct an error, or decide whether to slow down. These behaviors recruit executive function, sustained attention, working memory, sensory prediction, and motor calibration simultaneously. Automatic aerobic exercise can improve vascular and systemic biology, but CMI-specific recovery requires that the primed neural state be recruited by tasks that demand coordination, interference management, prediction, inhibition, and updating (Di Palma et al., 2025; Kantak and Winstein, 2012).

CMI should therefore be understood as a biological-behavioral interface rather than as an intervention that operates independently of peripheral, vascular, immune, and metabolic systems. Exercise can create a permissive physiological state through neurotrophic, vascular, immune-endocrine, and metabolic relays; CMI practice tests whether that state can be recruited during attentionally demanding action. The two components are potentially synergistic because exercise may increase readiness for adaptation, whereas CMI specifies the behavioral problem that the nervous system must solve.

The additional cognitive load imposed during CMI does not necessarily activate a completely separate pathway, but it can change the internal dose of the same exercise exposure. Adding working memory, inhibition, set shifting, obstacle negotiation, or response selection can increase autonomic demand, neurovascular demand, perceived effort, fatigue, and the need for network coordination. Thus, a dual-task intervention is not simply aerobic exercise plus a cognitive distraction; it is a modified biological and behavioral exposure whose safety and effectiveness depend on task selection, disease context, and preserved movement quality.

Dual-task interference models support this interpretation by showing that interference is shaped by resource competition, task prioritization, posture and gait phase, turning demands, and the executive content of the secondary task (Al-Yahya et al., 2011; Bayot et al., 2018). CMI should therefore be prescribed as a controlled therapeutic exposure: difficult enough to reveal the bottleneck, but not so difficult that the patient protects cognition by sacrificing gait safety or movement quality (Beurskens and Bock, 2012; Montero-Odasso et al., 2017; Mirelman et al., 2016).

Automaticity illustrates the distinction. Walking or cycling can improve cardiorespiratory and vascular support even when the movement is relatively automatic. However, many neurological disorders disrupt the economy of automatic control: gait becomes attentionally expensive, balance requires conscious oversight, and dual-task performance deteriorates. Under those conditions, recovery of mobility and recovery of cognition are not cleanly separable, because both depend on how efficiently the patient allocates attention and stabilizes movement under competing demands (Fritz et al., 2015; Leone et al., 2017; He et al., 2018).

Disease-specific pathology determines which form of CMI is most appropriate. After stroke, the emphasis may be on visuospatial attention, obstacle negotiation, divided attention during gait, or error-based relearning of paretic movement. In PD, the critical target may be cue responsiveness, set shifting, turning, freezing-related attentional control, and restoration or compensation of gait automaticity. In MS, the CMI dose must be fatigue-sensitive and often centered on processing speed, balance-attention coupling, and preservation of performance across repeated trials. In TBI, CMI exposure should be graded and symptom-limited, with attention to exertional tolerance, vestibulo-ocular load, headache, sleep, and autonomic response.

### 5.2 Ecological performance, adaptation, and learning

Error-based adaptation and sensorimotor prediction provide a second bridge between biology and behavior. Movement quality depends on anticipating consequences, detecting mismatches, updating internal models, and stabilizing the next attempt. These operations are computationally demanding and sensitive to fatigue, distraction, and disease-related slowing. A biologically active exercise session may therefore fail to produce meaningful recovery if subsequent practice is too passive, too simple, or too detached from decision-making. Task complexity matters because prediction, monitoring, and adaptation are part of the therapeutic target, not merely background demands.

Concrete CMI tasks should therefore be chosen for their mechanistic purpose. A memory-loaded walking task may be appropriate when delayed recall and route planning are limiting, whereas an inhibition-loaded turning task may better target impulsive stepping or freezing risk. A response-selection reaching task may be more relevant for upper-limb relearning after stroke than a verbal fluency task, while a fatigue-paced balance-attention protocol may be more appropriate in MS than a high-speed dual-task treadmill protocol. In TBI, the same cognitive-motor challenge may need to begin below the symptom threshold and progress only when recovery between sessions is stable (Krakauer, 2006; Krakauer and Mazzoni, 2011; Kleim and Jones, 2008).

This task-specificity should be reported explicitly in future studies. A trial should state whether the secondary task loads sustained attention, inhibition, working memory, verbal fluency, visuospatial scanning, or set shifting, because each cognitive demand can produce a different interference pattern and may stress a different disease-specific network (Bayot et al., 2018; Plummer et al., 2013). Without this specification, two interventions both labeled dual-task training may represent fundamentally different biological-behavioral exposures.

Ecologically valid cognition should also receive more weight in exercise trials. Patients often describe their limitation as difficulty walking while planning, talking while turning, sustaining posture while distracted, or maintaining performance when fatigued. These complaints reflect CMI in daily life. Motivation, affective burden, cognitive fatigue, perceived effort, and flow during therapy can influence how much useful practice is actually achieved. Engagement is therefore not a cosmetic feature; it can shape repetition quality, attentional investment, and durability of learning (Ottiger et al., 2021; Lang et al., 2009; Lohse et al., 2014; Kleim and Jones, 2008).

Seen through this lens, exercise should be described as a state-setting and capacity-building intervention rather than as a stand-alone cognitive drug. Its cognitive relevance becomes strongest when systemic pathway engagement, central responsiveness, and meaningful CMI demands are aligned. This framing explains why combined interventions can be stronger than isolated aerobic programs for some outcomes and why ecological endpoints may reveal benefit even when brief global cognitive tests do not.

The central implication is that CMI is unlikely to be equally effective across neurological populations when delivered as a uniform paradigm. Its added value over traditional exercise depends on matching the cognitive operation, motor context, intensity, feedback, and progression rule to the disease-specific mechanism that limits daily function. This disease-sensitive matching is what allows CMI to convert exercise-responsive biology into ecologically relevant recovery rather than merely adding difficulty to a conditioning program.

## 6 Disease-specific expression across neurological populations

### 6.1 Stroke

Stroke provides one of the clearest clinical reasons to study exercise-responsive CMI. Cognitive impairment after stroke is common, persistent, and strongly tied to mobility, self-care, and participation. Exercise is attractive in this setting because it can engage vascular responsiveness, neurotrophic signaling, mood, sleep, endurance, and motor relearning at the same time. Meta-analyses support a modest but meaningful effect of exercise on post-stroke cognition, especially when interventions are sustained, sufficiently dosed, and combined with behaviorally enriched practice (Oberlin et al., 2017; Li et al., 2022; Li et al., 2024; Wang et al., 2025).

Stroke also reveals why heterogeneity matters. Timing after onset, lesion topology, vascular reserve, diaschisis, neglect, aphasia, pre-existing cognitive burden, and deconditioning all shape the internal biological dose of training. A protocol that benefits one subgroup may underperform in another because the limiting factor differs, ranging from hypoperfusion and poor endurance to attentional instability or inefficient relearning. Biomarker and imaging studies suggest that aerobic training can alter neuroplasticity-related profiles after stroke, but network and cognitive outcomes remain variable (Ashcroft et al., 2022; Limaye et al., 2021).

Behaviorally enriched exercise appears especially relevant after stroke. Dual-task and cognitively loaded interventions may amplify benefit by requiring the recovering brain to allocate attention, update action plans, manage interference, and correct errors while movement unfolds. Meta-analytic evidence supports dual-task training for gait and balance, and comparative syntheses suggest that multimodal exercise may outperform routine care for post-stroke cognition when it is sufficiently specific and sustained (Fritz et al., 2015; He et al., 2018; Wang et al., 2025; Sun et al., 2025).

A stroke-specific CMI paradigm should therefore be anchored to the affected network and functional limitation. Patients with visuospatial neglect or attentional asymmetry may require walking or reaching tasks that explicitly force environmental scanning and obstacle avoidance, whereas patients with executive slowing may benefit from graded response-selection or set-shifting during gait. For upper-limb recovery, CMI may involve reaching while planning object use, correcting trajectory errors, or adapting to variable feedback. In each case, the purpose is not simply to make exercise harder, but to pair vascular and neurotrophic pathway engagement with the exact cognitive-motor operation required for relearning.

Stroke evidence specifically warns that CMI patterns can be heterogeneous, with some patients showing mainly motor deterioration and others showing mutual motor-cognitive costs during functional mobility (Plummer et al., 2013). This supports a precision approach in which neglect, aphasia, executive slowing, corticospinal tract integrity, and fall risk determine whether the added cognitive demand should emphasize scanning, response selection, balance allocation, or paretic-limb error correction.

### 6.2 Parkinson’s disease

PD presents a distinct case. The disorder is often framed in motor terms, yet cognitive impairment can involve executive function, attention, set shifting, visuospatial processing, and reduced automaticity from early stages onward. Exercise is mechanistically attractive in PD because it may influence dopamine-sensitive circuitry, frontostriatal efficiency, neurotrophic signaling, gait automaticity, cue responsiveness, and compensatory recruitment of cognitive control during movement (Petzinger et al., 2013; Duchesne et al., 2016; Kaagman et al., 2024; Ahlskog, 2011; Owen, 2004; Mak et al., 2017).

Clinical evidence in PD is encouraging but mixed. Updated meta-analyses indicate that exercise can improve global cognition or selected executive outcomes, with signals that mind-body, aerobic, and multimodal protocols may be useful, yet certainty remains limited and effect sizes vary by age, baseline cognition, medication exposure, motor phenotype, and dose (Kim et al., 2023; Folkerts et al., 2024; Chang et al., 2025; Huang and Zhang, 2025).

PD also exposes the importance of medication state and automaticity failure. A program may appear effective when patients are optimally medicated and able to recruit compensatory control, but produce smaller gains when freezing, fluctuations, postural instability, or cognitive fatigue dominate performance. The most clinically relevant outcome may therefore be not a generic cognitive score alone, but improved ability to maintain gait stability, cueing responsiveness, and cognitive control under competing demands.

A PD-specific CMI strategy should therefore focus on the failure of automatic control and the need to recruit compensatory cognitive strategies without overloading attention. Rhythmic auditory cueing, visual stepping targets, turning tasks with response inhibition, gait initiation under set-shifting demands, and freezing-related attentional control are examples of paradigms that connect exercise to the disease mechanism rather than applying a generic dual-task model. These tasks are most interpretable when medication state, freezing phenotype, fall risk, fatigue, and baseline executive function are documented.

This approach is supported by gait-cognition literature showing that executive control and attention become increasingly important when automatic locomotor control is compromised, and by PD-specific dual-task and cueing evidence indicating that external cues can partially compensate for impaired internal gait regulation (Yogev-Seligmann et al., 2008; Kelly et al., 2012; Nieuwboer et al., 2007). The key clinical risk is that the same cognitive challenge may improve cue use in one patient but destabilize freezing or postural control in another.

The evidence base also requires a cautious interpretation. Exercise studies in PD support cognitive and motor-cognitive benefits in selected domains, but CMI-specific mechanisms are not yet uniformly established. The plausible advantage of CMI over traditional exercise is that it directly trains the frontostriatal and attentional compensation required when automatic gait fails. The limitation is that excessive dual-task load can worsen gait, increase fall risk, or mask benefit if the patient lacks sufficient cognitive reserve or is tested in an unfavorable medication state (Mak et al., 2017; Beurskens and Bock, 2012; Montero-Odasso et al., 2017).

### 6.3 Multiple sclerosis

MS extends the model into a disease context dominated by inflammatory activity, demyelination, fatigue, heat sensitivity, and variable lesion distribution. Cognitive dysfunction commonly affects processing speed, attention, working memory, and dual-task capacity. Exercise is appealing because it can address deconditioning and mood while potentially modulating inflammatory tone, cardiorespiratory fitness, fatigue tolerance, and network efficiency (Sandroff et al., 2016; Lozinski and Yong, 2022).

Evidence in MS has shifted from broad uncertainty toward more differentiated conclusions. Recent meta-analyses and network comparisons suggest that selected exercise modalities can improve overall cognition or specific domains, although certainty still varies and superiority of one modality remains unsettled. Aerobic, balance-oriented, and multimodal interventions may all contribute, but response is conditioned by disability level, fatigue, relapse status, heat sensitivity, and the extent to which the program engages cognitive-motor demands (Gharakhanlou et al., 2021; Li et al., 2023; Bae et al., 2025; Su et al., 2025).

In MS, CMI should be framed around fatigue-sensitive processing rather than around maximal task difficulty. A disease-matched paradigm may combine balance or gait practice with processing-speed, divided-attention, or working-memory demands while monitoring heat sensitivity, perceived fatigue, recovery between sessions, and next-day symptom burden. The aim is to improve consistency of cognitive-motor performance under realistic load, not to force high-intensity cognitive challenge in a system vulnerable to fatigability and fluctuating disability.

MS-specific evidence supports this conservative framing because cognitive-motor interference is well documented, but its correlates and consequences vary with disability, fatigue, balance impairment, and cognitive reserve (Wajda and Sosnoff, 2015; Learmonth et al., 2017). Dual-task training may improve dynamic balance and functional mobility in MS, yet prediction of future falls from dual-task testing remains insufficiently consistent to justify a one-size-fits-all clinical algorithm (Martino Cinnera et al., 2021; Abou et al., 2022).

The potential advantage over traditional exercise is that CMI targets the everyday problem many patients report: maintaining gait, balance, or manual performance while thinking, planning, or responding under fatigue. The current evidence supports exercise-related cognitive benefit in MS, but the optimal CMI dose, task type, cooling strategy, and fatigue-management algorithm remain incompletely defined. This uncertainty should guide trial design toward symptom-aware dual-task endpoints rather than toward uniform prescriptions (Motl et al., 2017; Benedict et al., 2020; Beurskens and Bock, 2012).

### 6.4 Traumatic brain injury

TBI introduces a different translational problem because injury severity, chronicity, symptom profile, sleep quality, autonomic stability, headache burden, vestibulo-ocular symptoms, and exertional tolerance vary enormously. Exercise is biologically plausible when framed around cerebral perfusion, mood regulation, autonomic recalibration, sleep, and progressive return to cognitively demanding activity. Clinical evidence remains promising but not definitive because trials are small, heterogeneous, and methodologically diverse (Vanderbeken and Kerckhofs, 2017; Alashram, 2026; De Luigi et al., 2023).

Randomized and proof-of-concept studies in chronic TBI suggest that moderate aerobic training can improve memory or neurocognitive performance in some individuals, while others show little measurable change despite acceptable tolerability. This variability is clinically informative. Symptom provocation, vestibulo-ocular burden, sleep disruption, headache, and autonomic dysregulation may alter how much effective training is possible and whether a given protocol is experienced as restorative or destabilizing (Chin et al., 2015; Gladstone et al., 2019; Ding et al., 2021; Wender et al., 2021).

For TBI, CMI should be introduced as a graded exposure to cognitive-motor demand rather than as immediate high-complexity dual-task training. A practical sequence may begin with symptom-limited aerobic work, then add low-load attention or response-selection tasks, and only later progress toward divided attention, vestibulo-ocular complexity, or real-world navigation. This progression is especially important when headache, dizziness, sleep disruption, visual motion sensitivity, or autonomic symptoms determine exercise tolerance.

Concussion and mild TBI studies support this staged logic because gait abnormalities may be more visible under dual-task or complex gait conditions than during simple walking, and symptom-limited subthreshold aerobic exercise has randomized-trial support in sport-related concussion (Howell et al., 2013; Fino et al., 2018; Leddy et al., 2019). For rehabilitation, the practical implication is that cognitive exertion, vestibulo-ocular load, and aerobic load should be progressed together only when symptoms return to baseline.

The plausible added value of CMI in TBI is that it bridges physiological recovery with return to cognitively demanding activity. Traditional aerobic training may improve tolerance and autonomic regulation, but patients often fail when exertion is combined with attention, memory, environmental motion, or multitasking. CMI paradigms can therefore test and train the combined load that matters for community participation, provided that symptom thresholds, recovery time, and safety are treated as dosing variables rather than as secondary observations (Griesbach, 2011; Ponsford et al., 2012; Esterov and Greenwald, 2017).

Across all four conditions, disease-specific biology modifies every stage of the pathway from exercise exposure to cognitive-motor outcome. Shared mechanisms exist, but translation is never disease-neutral. Stroke, PD, MS, and TBI differ in vascular reserve, neurochemical state, immune activity, energetic limits, symptom provocation, and ecological task constraints. A uniform prescription may therefore obscure rather than reveal the true therapeutic potential of exercise. The main disease-specific modifiers that help explain this heterogeneity are summarized in Figure 2. Complementary disease-specific comparisons are summarized in Table 3.

**TABLE 3:** Disease-specific expression of exercise-related cognitive-motor adaptation across major neurological populations.

Condition	Typical cognitive profile	Dominant biological or pathophysiological modifiers	Exercise modalities with strongest current support	Most relevant technology-enabled or multimodal adjuncts	Frequent sources of inconsistency	Clinically important moderators and cautions
Stroke	EF, attention, processing speed, dual-task instability, variable memory involvement	Lesion topology, recovery stage, vascular reserve, diaschisis, deconditioning	Aerobic training, multimodal exercise, and dual-task enriched practice show the strongest current support (Li et al., 2022; Li et al., 2024; Wang et al., 2025). CMI examples include obstacle negotiation, divided attention during walking, visuospatial scanning, and error-based upper-limb relearning.	VR, robotics for high-dose practice, wearables, and selected NIBS pairing may be useful adjuncts (He et al., 2018; Laver et al., 2025).	Mixed timing after stroke, heterogeneous cognitive outcomes, variable intensity reporting	Early tolerance, cardiovascular status, aphasia, neglect, fatigue, task-specific relearning needs
PD	EF, set shifting, attention, visuospatial deficits, reduced automaticity	Dopaminergic state, frontostriatal dysfunction, gait automaticity failure, freezing vulnerability	Aerobic exercise, multimodal programs, mind-body exercise, and cognitively loaded gait work appear most promising (Kim et al., 2023; Folkerts et al., 2024; Huang and Zhang, 2025). CMI examples should emphasize cueing, set shifting, turning, freezing-related attentional control, and gait automaticity.	Cueing systems, VR, gait-focused wearables, and exercise plus NIBS may be useful in selected cases (Intzandt et al., 2018; Xia et al., 2025).	Medication timing, age, baseline cognition, dose variability, motor phenotype differences	Monitor fluctuations, freezing, cognitive fatigue, fall risk, medication state during testing Avoid assuming that higher dual-task complexity is beneficial if it worsens gait safety or freezing.
MS	Processing speed, attention, WM, cognitive-motor interference, fatigue-sensitive performance	Inflammation, demyelination, heat sensitivity, fluctuating disability, network inefficiency	Aerobic exercise, balance-oriented programs, and multimodal combined approaches currently have the strongest support (Gharakhanlou et al., 2021; Li et al., 2023; Bae et al., 2025; Su et al., 2025). CMI examples should be fatigue- and heat-aware, with processing-speed and balance-attention demands progressed conservatively.	Wearables, VR for graded complexity, remote monitoring, and symptom-adapted dual-task training may be especially relevant (Gharakhanlou et al., 2021; Su et al., 2025).	Small samples, mixed disability levels, fatigue effects, inconsistent domain-specific endpoints	Respect heat sensitivity, symptom fluctuation, fatigue burden, relapse status, adherence limits Use recovery-aware pacing; next-day fatigue is a dosing signal, not merely an adherence issue.
TBI	Attention, memory, EF, slowed processing, symptom-sensitive task breakdown	Injury heterogeneity, autonomic instability, sleep disruption, exertional intolerance, headache burden	Graded aerobic training, symptom-limited conditioning, and selected multimodal programs appear most relevant (Chin et al., 2015; Wender et al., 2021; Alashram, 2026). CMI examples should be graded and symptom-limited, especially when vestibulo-ocular, headache, sleep, or autonomic symptoms are present.	Wearables, VR in selected patients, active rehabilitation frameworks, and cautious NIBS use may support translation (Bonanno et al., 2022; De Luigi et al., 2023; Zaninotto et al., 2019).	Small trials, mixed severity, chronicity differences, symptom provocation during training	Progress slowly, monitor symptoms closely, consider vestibular burden, sleep, and autonomic response Progress only when symptoms return to baseline and combined cognitive-motor load is tolerated.

EF, executive function; MS, multiple sclerosis; NIBS, non-invasive brain stimulation; PD, Parkinson’s disease; TBI, traumatic brain injury; VR, virtual reality.

These comparisons also clarify why CMI should not be prescribed as a single standardized dual-task package. Stroke may require lesion- and attention-specific relearning, PD may require cue-dependent compensation for automaticity failure, MS may require fatigue- and heat-aware cognitive-motor pacing, and TBI may require symptom-limited progression under autonomic and vestibulo-ocular monitoring. The shared principle is alignment between internal biological dose and the cognitive-motor operation that limits daily function; the clinical implementation differs because the disease mechanisms differ (Ahlskog, 2011; Motl et al., 2017; Silverberg et al., 2020).

## 7 Technology-enabled and personalized rehabilitation implications

### 7.1 Exercise prescription, internal load, and timing

If exercise is to be used as a cognitively relevant neurorehabilitation tool, prescription must be guided by more than safety and motor goals (Table 4). The key translational question is how modality, intensity, duration, sequencing, and task context can align biological pathway engagement with behavioral demand. Aerobic exercise is especially relevant for cardiorespiratory fitness, cerebral blood flow, vascular shear stress, lactate dynamics, and neurotrophic responses. Resistance exercise contributes through muscle quality, endocrine signaling, postural reserve, fatigue resistance, and tolerance for task practice (Rodríguez-Gutiérrez et al., 2023; Oporto-Colicoi et al., 2025). In stroke, aerobic and multimodal approaches may be particularly relevant for vascular reserve and relearning; in PD, gait-cognition and cueing-based programs may target automaticity; in MS, fatigue-sensitive aerobic, balance, and multimodal programs may be most appropriate; and in TBI, graded symptom-limited aerobic exposure is often central.

**TABLE 4:** Translational framework for exercise prescription in cognitive-motor neurorehabilitation.

Rehabilitation objective	Preferred exercise logic or modality emphasis	Target internal load or dosing principle	Best timing or context of use	Key monitoring variables	Mechanistic emphasis	Likely cognitive-motor targets	Practical cautions
Improve global cognitive-motor endurance	Aerobic or multimodal conditioning with progressive overload is generally appropriate for this aim (Erickson et al., 2019; Wang et al., 2025).	Sufficient internal challenge without symptom destabilization	Chronic and subacute phases when tolerance allows consistent repetition	Heart rate, perceived exertion, symptom response, session adherence	Vascular responsiveness, metabolic support, general plasticity readiness	Attention during sustained movement, processing efficiency, participation tolerance	Avoid equating external workload with biological dose
Reduce dual-task cost during gait and balance	Dual-task walking, balance plus cognitive challenge, and multicomponent sessions are most aligned with this objective (Fritz et al., 2015; He et al., 2018). Select the cognitive load deliberately: attention, inhibition, set shifting, memory, or visuospatial scanning should match the functional deficit.	Moderate load with preserved task quality and attentional engagement	When gait is safe enough to add cognitive complexity	Dual-task cost, gait variability, balance errors, attentional drift	CMI, attentional allocation, action selection, network coordination	Automaticity, interference management, real-world mobility	Increase complexity gradually to prevent unsafe trade-offs Do not use the same dual-task paradigm across diagnoses without adapting task type, symptom threshold, and safety constraints.
Support EF during locomotor adaptation	Aerobic priming followed by task-switching or response-inhibition practice is a plausible strategy (Leone et al., 2017; Chang et al., 2025). Disease-matched examples include cue-response switching in PD and visuospatial response selection after stroke.	Priming session plus cognitively demanding movement block	Useful when planning, set shifting, or cueing deficits limit mobility	Task accuracy, reaction time, cue dependence, fatigue trajectory	Frontoparietal engagement, metabolic readiness, adaptive control	Set shifting, attentional control, gait adaptation	Do not separate biological priming from the target behavior by long delays
Enhance upper-limb cognitive-motor learning	Task-oriented practice, robotics-assisted repetition, and variable feedback may best support this aim (Scott and Dukelow, 2011; Morone et al., 2020).	High repetition with preserved precision and manageable cognitive load	After basic movement is available and practice dose is a limiting factor	Error rate, movement smoothness, task completion, patient engagement	Error-based adaptation, sensorimotor prediction, repetition-dependent plasticity	Planning during reach, online correction, visuomotor control	Excessive assistance can reduce learning value if challenge is too low
Manage fatigue-sensitive rehabilitation in MS or TBI	Symptom-limited aerobic or multimodal sessions with pacing are usually most appropriate (Gharakhanlou et al., 2021; Leddy et al., 2023; Alashram, 2026).	Submaximal internal load, flexible progression, recovery-aware scheduling	Fatigue-dominant or exertion-sensitive phases	Perceived fatigue, symptom flare, sleep, autonomic response, next-day function	Energetic efficiency, inflammatory moderation, tolerance building	Sustained attention, consistency under load, reduced cognitive fatigability	Heat, headache, dizziness, and delayed symptom worsening require close tracking CMI progression should stop or regress when cognitive load destabilizes symptoms, gait, balance, or next-day recovery.
Bias plasticity during selected technology-assisted sessions	Exercise paired with VR, wearables, robotics, or NIBS should be matched to the specific translational target (Tieri et al., 2018; Laver et al., 2025; Xia et al., 2025).	Dose matched to the specific translational purpose of the technology	When ecological challenge, feedback density, or network biasing is needed	Behavioral metrics, sensor data, tolerability, timing relative to exercise	Context shaping, repetition scaling, ecological monitoring, network modulation	Transfer to daily tasks, adaptive performance, task-specific gains	Technology should solve a defined problem, not replace therapeutic logic

CMI, cognitive-motor integration; MS, multiple sclerosis; NIBS, non-invasive brain stimulation; TBI, traumatic brain injury; VR, virtual reality.

A practical CMI prescription begins by selecting the cognitive operation to be trained during movement. Attention tasks may emphasize sustained monitoring, target detection, or divided attention; inhibition tasks may require stopping, turning, or suppressing an automatic response; set-shifting tasks may require alternating rules or cue-response mappings; working-memory tasks may involve route recall, object sequences, or delayed responses. The cognitive domain should be selected because it matches the patient’s functional limitation, not because it is convenient to add to exercise.

Progression should then move from single-task safety to dual-task accuracy, environmental complexity, variable feedback, and real-world transfer. Task complexity can be increased by manipulating speed, sensory clutter, obstacle density, response uncertainty, memory load, feedback delay, or the number of simultaneous rules. However, complexity should be advanced only when gait, balance, reaching quality, symptom response, and recovery remain acceptable; otherwise, the intervention risks training compensation, instability, or fatigue rather than adaptive CMI.

Intensity should be interpreted as an internal biological construct rather than a simple external workload. Higher intensity can produce larger lactate responses and acute biomarker shifts, but intensity is not therapeutic by itself. A useful dose is one that engages the intended pathway without overwhelming autonomic control, provoking symptoms, degrading movement quality, or reducing learning opportunities. In stroke this may require cardiovascular monitoring and gradual progression; in PD it should account for medication state and freezing risk; in MS it should respect fatigue and heat sensitivity; and after TBI it should remain symptom-limited and closely monitored (Zoladz et al., 2014; Leddy et al., 2018; Leddy et al., 2023; Wang et al., 2025).

Stroke and PD illustrate why diagnosis-specific paradigms are necessary. A stroke patient with visuospatial neglect may benefit from obstacle negotiation with scanning demands, whereas a PD patient with freezing may require rhythmic cueing, anticipatory turning, and response-inhibition tasks timed to medication state. These are not interchangeable dual-task prescriptions. The same cognitive label, such as attention, can require different motor contexts, cues, progression rules, and safety thresholds depending on whether the limiting mechanism is focal injury, frontostriatal automaticity failure, demyelinating fatigue, or diffuse post-traumatic dysregulation.

Timing also matters. Early after stroke, exercise may help restore vascular responsiveness and create opportunities for use-dependent relearning, but medical stability and fatigue limit how aggressively it can be delivered. In PD, ongoing exercise may support compensatory control and maintain function under progressive network stress. In MS, dosing should respect fatigue cycles and inflammatory activity. After TBI or concussion-related syndromes, graded aerobic exposure can be useful when symptom monitoring is explicit and progression is paced according to individual tolerance (Leddy et al., 2018; Leddy et al., 2023; De Luigi et al., 2023).

Personalization therefore requires more than choosing an exercise type. Baseline fitness, lesion burden, medication state, heat sensitivity, fatigue, pain, sleep, autonomic stability, and cognitive load tolerance all change the meaning of a session. Monitoring should combine external workload with internal load, symptom response, movement quality, and ecologically relevant performance, because the same treadmill speed, cycling wattage, or virtual task can represent very different biological and cognitive exposures across patients.

In practice, CMI integration can be conceptualized as four linked decisions: first, define the biological exposure that is tolerable; second, select the movement behavior that matters clinically; third, choose the cognitive demand that reflects the patient’s limitation; and fourth, monitor whether the combined task preserves quality while challenging adaptation. VR, robotics, wearables, and NIBS should then be used only when they improve one of these decisions, such as by increasing contextual salience, scaling repetition, measuring ecological transfer, or biasing a network during active practice.

### 7.2 Multimodal integration with dual-task training, VR, robotics, wearables, and NIBS

The clinical promise of exercise becomes stronger when multimodal tools are selected for a defined translational purpose rather than added as generic enhancements. Dual-task training intensifies cognitive-motor demand and reveals whether biological readiness is translating into interference management. Virtual reality manipulates sensory complexity, salience, reward, contextual variability, and error opportunities. Robotics can scale repetition, assistance, resistance, timing, and measurement precision. Wearables capture ecological activity, gait variability, symptom fluctuation, and dual-task cost outside the clinic. NIBS may help bias selected networks during or around active training, although effects remain protocol-dependent and should not be assumed to generalize across diagnoses (Semprini et al., 2018; Tieri et al., 2018; Bonanno et al., 2022; Laver et al., 2025; Xia et al., 2025).

The relevance of each technology differs by disease. Stroke may benefit from robotics and VR when repetition, feedback, and task-specific relearning are limiting. PD may benefit from cueing, gait-focused wearables, VR, and combined exercise-NIBS approaches when automaticity and freezing are central. MS may benefit from remote monitoring, adaptive pacing, VR-based complexity, and fatigue-sensitive dual-task progression. TBI may benefit from wearables, graded exposure, symptom monitoring, and cautious use of immersive environments when vestibular symptoms or headache are prominent.

Technology should therefore be integrated with biomarkers, vascular biology, immune-endocrine context, and behavioral targets. A wearable outcome is most useful when it tests whether improved clinic performance transfers to daily mobility. A VR task is most useful when it manipulates contextual complexity relevant to the target behavior. NIBS is most defensible when paired with active practice and a clear network hypothesis. Robotics are most valuable when they solve a dosage or precision problem rather than simply increasing passive repetition.

Personalized technology-enabled rehabilitation should combine external workload, internal physiological load, symptom response, task quality, and ecological behavior. The same nominal intervention can be a vascular challenge in one patient, a fatigue-provoking exposure in another, and a cognitively meaningful CMI task in a third. Next-generation trials should predefine which mechanism they intend to engage and select the technology that best tests or supports that mechanism.

These technologies should not be viewed as competing innovations. Each solves a different translational problem: dual-task paradigms intensify cognitive-motor demand; VR manipulates context and salience; robotics increase precision and repetition; wearables capture ecological response; and NIBS may bias plasticity. Their value depends on mechanism-matched use, disease-specific constraints, and whether they improve transfer to meaningful daily function.

Mechanism matching is the critical distinction between useful technology and technological decoration. Neurocognitive robot-assisted hand therapy after stroke illustrates how robotic precision can be paired with somatosensory and cognitive task demands rather than with repetition alone (Ranzani et al., 2020). Similarly, wearable sensors can extend CMI assessment beyond the clinic by capturing gait variability, fall-risk features, and real-world mobility patterns in neurological populations, but those data must be interpreted within the clinical question being tested (Bonanno et al., 2025).

## 8 Discussion

### 8.1 Why compelling biology often translates into modest or inconsistent cognitive gains

The central conclusion of this review is that motor exercise can influence cognition through biologically credible pathways, but those pathways become clinically meaningful only when peripheral signals, central translation, and behavioral demands align. BDNF, IGF-1, lactate, irisin-related signaling, immune modulation, vascular adaptation, and endocrine-metabolic relays all offer plausible routes through which exercise may alter the conditions for brain adaptation. None of them, alone, establishes recovery. The key question is whether pathway engagement is linked to central physiology and then to ecologically useful CMI.

The strongest interpretation is not that these biological systems are irrelevant to recovery, but that they are insufficient as isolated surrogate endpoints. A vascular improvement may support attention and processing efficiency, an immune shift may reduce fatigue-promoting inflammatory tone, and metabolic adaptation may improve tolerance for sustained practice. Nevertheless, recovery requires evidence that these changes reach a central system, persist long enough to influence practice, alter network or neurophysiological function, and appear as measurable behavior in the task domain that matters to the patient.

This caution should not be read as denying meaningful biomarker-brain associations. Exercise-induced increases in BDNF have been associated with hippocampal growth and memory benefit in older adults, and BDNF has been proposed as a mediator of executive function improvement after exercise training (Erickson et al., 2011; Leckie et al., 2014). The point is epistemological: an association between a biomarker and a neural or behavioral outcome strengthens mechanistic plausibility, but it does not allow every peripheral increase to be interpreted as central repair or functional recovery.

The same logic applies to immune, vascular, and metabolic findings. Evidence that exercise improves cerebrovascular function, inflammatory balance, or substrate regulation strengthens the mechanistic plausibility of cognitive benefit, but it does not specify whether the patient has regained dual-task control, gait adaptability, planning during movement, or daily participation. What is missing is not biological plausibility; it is a pathway-linked chain of evidence from peripheral response to central translation and then to disease-appropriate CMI behavior.

Heterogeneity is a major reason why effects remain modest or inconsistent. Exercise is often treated as a single class even though walking, interval training, resistance work, balance training, exergaming, dual-task practice, and combined programs impose different physiological and cognitive demands. Populations are equally heterogeneous. Trials frequently combine individuals who differ in chronicity, lesion burden, cognitive status, medication state, fatigue, symptom tolerance, and baseline fitness, then rely on one outcome to summarize all of them. This design can obscure mechanism-specific benefit (Voss et al., 2013; Roig et al., 2013; Dobkin, 2009).

A peripheral biomarker increase is therefore not sufficient by itself to infer neural recovery. Biomarkers can show that a session engaged a biologically relevant pathway, but they do not establish tissue source, persistence, central uptake, network-level consequence, or behavioral use. This limitation can be addressed by pairing peripheral markers with neurophysiological, perfusion, imaging, and ecological behavioral readouts selected according to the hypothesized pathway, rather than by adding more markers without a clear mechanistic question.

This point is especially important when improvements occur at different time scales. Acute lactate, BDNF, or IL-6 responses may last minutes to hours; vascular and fitness adaptations may evolve over weeks; disease symptoms and learning may fluctuate across days; and ecological participation may require months of repeated transfer. Trials that measure only one point in this chain risk concluding either that exercise has no cognitive value or that a biomarker proves recovery, when the real issue is that the wrong level of the pathway was measured at the wrong time (Fleming and Powers, 2012; Cramer et al., 2011; Dobkin, 2009).

This is also a surrogate-endpoint problem. A biomarker can indicate normal biological processes, pathogenic processes, or response to an intervention, but a surrogate endpoint must reliably substitute for a clinical endpoint, which requires a stronger evidentiary standard than pathway engagement alone (Biomarkers Definitions Working Group, 2001). In exercise-CMI rehabilitation, peripheral markers should therefore be treated as mechanistic anchors unless they are empirically linked to central translation and clinically meaningful behavior.

Exercise alone should not be expected to improve cognition uniformly across all neurological populations. Exercise may prime the system, but priming is not equivalent to learning. Task structure, contextual richness, attentional demand, error opportunities, and ecological practice likely determine whether a favorable biological state is recruited into durable change. Improved automaticity, reduced dual-task cost, greater error tolerance, and more stable performance under cognitive load may therefore be more sensitive indicators of benefit than isolated global cognitive scores.

Disease biology matters as much as exercise biology. Stroke, PD, MS, and TBI are not interchangeable models of dysfunction. Their inflammatory profiles, vascular reserve, network architecture, energetic limits, medication contexts, and symptom provocation thresholds differ. The same external workload can therefore carry different internal biological meanings. This perspective argues for individualized dosing based on internal load, task relevance, disease mechanism, and ecological target rather than routine transplantation of protocols across disorders (Saunders et al., 2020; Motl et al., 2017; Silverberg et al., 2020).

### 8.2 What the next generation of studies should measure

The next generation of studies should be built around integrated mechanistic trials rather than broad claims that exercise is good for cognition. Trials need to specify the pathway they intend to engage, define exercise dose in physiological as well as external terms, and select outcomes that match the hypothesized mechanism. A study centered on lactate-responsive signaling should not rely only on a delayed global cognitive screen. A study centered on CMI should include dual-task behavior, gait adaptability, upper-limb cognitive-motor performance, contextual complexity, or wearable-derived real-world behavior.

Measurement timing also requires precision. Acute biomarker shifts are often captured immediately after training, whereas cognitive or CMI outcomes may be assessed at different biological and behavioral time scales. Intermediate outcomes such as TMS excitability, paired associative stimulation response, cerebral perfusion, neurovascular reactivity, functional network dynamics, task-based electroencephalography, or cognitive-motor task behavior may provide a clearer bridge between session biology and longer-term function. These tools should be selected to test a mechanistic hypothesis rather than added as technological ornaments.

Outcome ecology should move closer to daily life. Many patients describe their cognitive limitation as difficulty walking while planning, talking while turning, or sustaining goal-directed movement when fatigued and distracted. Those complaints capture the lived expression of CMI. Trials that include dual-task walking, adaptive balance tasks, upper-limb cognitive-motor testing, home mobility, and sensor-derived behavioral data are more likely to detect meaningful change than trials relying only on brief clinic-based screening tools (Fritz et al., 2015; Leone et al., 2017; Ottiger et al., 2021).

The biomarker agenda should expand, but with discipline. BDNF, IGF-1, irisin, and lactate remain important, yet they should not be treated as interchangeable proxies for plasticity. Vascular endothelial growth factor (VEGF) may help index angiogenic and endothelial components of exercise adaptation; IL-6 can reflect acute myokine signaling and chronic inflammatory context, but these meanings must be separated; cathepsin B has been linked to running-induced memory-related signaling; kynurenine pathway modulation provides a muscle-brain route through which exercise may influence neuroactive metabolites; glycosylphosphatidylinositol-specific phospholipase D1 (GPLD1) has been proposed as a liver-derived mediator of exercise-related brain benefit; and beta-hydroxybutyrate may link metabolic state to BDNF-related transcription (Agudelo et al., 2014; Moon et al., 2016; Sleiman et al., 2016; Horowitz et al., 2020; Zanganeh et al., 2026). Each candidate marker should be tied to a hypothesized pathway, sampling window, central readout, and behavioral endpoint.

A more useful biomarker strategy is therefore pathway-matched rather than list-based. VEGF is most informative when linked to vascular or perfusion readouts and to tasks that depend on sustained neural supply during movement. IL-6 should be interpreted differently as an acute myokine signal versus a marker of chronic inflammatory burden, and its relevance to CMI should be tested through fatigue-sensitive performance. Cathepsin B, kynurenine-pathway markers, GPLD1, and beta-hydroxybutyrate are valuable only when their sampling windows, tissue sources, central correlates, and behavioral endpoints are specified in advance. This approach turns candidate markers into testable translational hypotheses rather than adding more variables to an already heterogeneous field.

Several practical principles follow. Exercise prescriptions for cognition should be individualized by disease context, symptom burden, vascular reserve, immune-metabolic state, internal load, and task relevance. Multimodal programs should be designed so that biological pathway engagement and cognitive-motor challenge coincide. Technologies should be selected for the translational problem they solve. Most importantly, the field should stop asking whether exercise works for cognition in the abstract and start asking for whom, under which disease constraints, at what internal biological dose, through which pathway, paired with what behavioral task, and toward which ecologically relevant outcome.

The decisive design principle is alignment. Exercise dose should be aligned with the pathway marker; the marker should be aligned with a central readout; the central readout should be aligned with the CMI task; and the CMI task should be aligned with an ecological outcome. Only this chain can determine whether exercise-responsive biology has become clinically meaningful cognitive-motor recovery.

This alignment should be prespecified rather than reconstructed after results are known. The most defensible trials will define the intended biological pathway, select a marker with an appropriate sampling window, choose a central readout that matches that pathway, embed a disease-specific CMI task, and test whether the effect transfers to daily function. This structure would convert the manuscript’s integrative model into an experimentally falsifiable rehabilitation framework.

## 9 Conclusion

Motor exercise should no longer be regarded as a peripheral intervention in neurorehabilitation. It is an exposure capable of reshaping the biological conditions under which the injured or diseased brain learns, adapts, and coordinates cognition with movement. Peripheral mediators, vascular and immune relays, central plasticity, and systems-level reorganization all support this view, but their clinical value depends on translation into meaningful CMI.

The available evidence does not support simplistic claims. Cognitive benefit is plausible and sometimes measurable, yet it remains heterogeneous and context dependent. Disease biology, symptom burden, baseline fitness, medication state, internal load, and behavioral embedding all influence whether exercise becomes restorative, compensatory, or merely tolerable. Biomarkers can inform this process, but they cannot stand in for behavior.

For clinical practice, the most useful message is that cognitive and motor recovery should be trained as interacting dimensions of the same adaptive system. For research, the priority is to connect exercise prescription, mechanistic readouts, technology-enabled monitoring, and ecologically valid cognitive-motor outcomes within the same trial logic. From muscle to mind, the strongest path forward is not reductionist. It is integrative, disease-sensitive, and behaviorally precise.

The decisive translational step is therefore not the demonstration that exercise changes a molecule, a perfusion measure, or a network index in isolation. It is the demonstration that a disease-appropriate biological exposure can be coupled to a disease-appropriate CMI task and expressed as safer, more adaptable, and more durable behavior in daily life.

In practical terms, biological activation could be treated as the beginning of the translational chain, whereas disease-specific CMI transfer is the critical test that the chain has reached behavior.
